# Evaluation of the stability of immediately reinserted orthodontic miniscrews using a novel bioactive adhesive composite: an in vivo animal study

**DOI:** 10.1186/s40510-025-00595-x

**Published:** 2025-11-03

**Authors:** Neda Babanouri, Fatemeh Hajipour, Mohammad Mokhtarzadegan, Nader Tanideh

**Affiliations:** 1 Orthodontic Research Center, School of Dentistry, Shiraz University of Medical Science, Shiraz, Iran; 2 Postgraduate Student Dept. of Orthodontics, School of Dentistry, Shiraz University of Medical Sciences, Shiraz, Iran; 3 Department of Material Science and Engineering, Tehran University, Tehran, Iran; 4 Department of Pharmacology, School of Medicine, Shiraz University of Medical Sciences, Shiraz, Iran

**Keywords:** Orthodontic miniscrew, Miniscrew stability, Reinsertion, Chitosan, Hydroxyapatites, Beta-tricalcium phosphate, Postbiotic, Rats

## Abstract

**Background:**

To evaluate the effectiveness of a novel bioactive adhesive composite—comprising chitosan (CS), nanohydroxyapatite (nHA), β-tricalcium phosphate (β-TCP), and postbiotics—in enhancing the stability of orthodontic miniscrews immediately reinserted following failure in the same insertion site using a new miniscrew in an in vivo rat femur model.

**Methodology:**

Composite adhesives were prepared in two distinct forms: foam and paste. Orthodontic miniscrews were placed into the left femurs of 28 male Sprague‒Dawley rats, and a small hole was drilled 15 mm distal to the miniscrew site. Orthodontic force was applied via a 100-gram Ni‒Ti closed coil spring attached between the miniscrew and the drilled hole. After 4 weeks, the rats were divided into four groups: Group 1 (4-week control), Group 2 (8-week control), Group 3 (paste adhesive), and Group 4 (foam adhesive). Group 1 rats were euthanized to assess primary miniscrew stability. Groups 2, 3, and 4 underwent a second surgery in which miniscrews were carefully removed and reinserted without adhesive, with paste-form adhesive, and with foam-form adhesive, respectively. After an additional 4 weeks, miniscrew stability was evaluated through histological analysis and mechanical pull-out testing. The data were analyzed via one-way ANOVA followed by Duncan’s post hoc test. Normality was confirmed with the Shapiro‒Wilk test, and effect sizes (Eta Squared) were calculated due to the small sample size.

**Results:**

Reinsertion without adhesive reduced the stability of miniscrews. However, when adhesive was used during reinsertion, the maximum force required to extract the miniscrew from the surrounding bone significantly increased, with the foam group demonstrating the highest pull-out strength (PS). Compared with the control group, the paste and foam groups presented greater mean bone‒implant contact (BIC) and bone volume (BV) values, although these differences were not statistically significant (*p* > 0.05). Effect size analysis revealed large differences in the clinical effects of BV and PS between the control groups and the adhesive-treated groups.

**Conclusions:**

The bioactive adhesive composite can improve the primary stability of orthodontic miniscrews immediately reinserted into the same site after failure. The findings suggest that such adhesives may serve as a temporary adjunct to support anchorage continuity, warranting further in vivo evaluation before clinical application.

## Background

Miniscrews, as temporary anchorage devices (TADs), are widely used in orthodontics to provide resistance against undesired tooth movement [1]. Their rapid and easy insertion into bone makes them an efficient method for achieving stable anchorage in the treatment of dental and skeletal malocclusions without relying on patient compliance [2]. Despite their advantages, miniscrews may experience unforeseen loosening during treatment, potentially jeopardizing the entire orthodontic treatment plan [3–5]. In such cases, the options include either reinserting the miniscrew or modifying the treatment plan with alternative anchorage methods [6]. Reinsertion can be performed at the same site or in an adjacent area [2, 7, 8]. However, anatomical challenges, such as severe pneumatization of the maxillary sinus, a narrow interradicular space, insufficient attached gingiva, or unfavorable frenal attachment, may interfere with reinsertion in adjacent areas [9]. When reinsertion at the same site is necessary, a healing period of 3–6 months is typically required for bone regeneration [10–12]. However, studies have shown that the success rate of secondary mini-screw insertion is generally lower. Notably, Uesugi et al. reported a significant decrease in the success rates of mini-screws reinserted into the maxilla [2]. Also, they found no significant difference in failure rates between reinsertion at the same site and reinsertion at a different location [6].

In the field of dental implants, efforts have been made to enhance the primary stability of implants by investigating various materials and techniques [13, 14]. Andersen et al. demonstrated that adhesive cements have the potential to enhance the primary stability of various dental implant designs, as evidenced by an increase in the removal torque after the cement was applied [15]. In orthodontics, studies have been conducted to improve the stability of miniscrews [16, 17]. However, research specifically focused on enhancing the stability of reinserted miniscrews remains limited, and only one study has investigated the use of adhesive cements as a bone-filling material to augment stability following reinsertion [8]. Although β-tricalcium phosphate was used as the only material in that study and showed some potential, it is associated with several significant limitations. Owing to its porous structure, its mechanical strength is relatively low, which makes it unsuitable for withstanding the forces applied immediately after miniscrew reinsertion [18, 19]. Additionally, its powder form complicates its practical application in clinical settings. These limitations emphasize the need for more effective materials that can better meet the demands of orthodontic treatments.

In this context, the promising outcomes of hydroxyapatite (HA)/ β-tricalcium phosphate (β-TCP)/chitosan (CS) composites in bone tissue engineering provide a basis for their potential use in stabilizing loosened miniscrews [20]. Immediate reinsertion of loosened miniscrews in orthodontic treatment offers advantages by reducing delays and preserving treatment mechanics. Hence, this study aimed to develop a composite adhesive with two distinct forms designed to stabilize loosened miniscrews upon immediate reinsertion and to evaluate its effectiveness in maintaining stability.

## Methods

### Adhesive preparation

A 2 wt% chitosan solution was prepared by dissolving chitosan (Green Exir Biomaterials, Shiraz, Iran) in 0.2 M acetic acid and stirring the mixture for 2 h to ensure complete homogenization [21]. The solution was then filtered to remove air bubbles. Subsequently, the following components as mentioned in Table 1 were sequentially added to the chitosan hydrogel: (1) a postbiotic derived from Lactobacillus (3% v/v), (2) nanohydroxyapatite (nHA, Apatech, Yazd, Iran; 6% w/v), and (3) β-tricalcium phosphate (β-TCP, Merck, Darmstadt, Germany; 21% w/v) [20, 22, 23]. Each component was incorporated with at least 1 h of stirring to maintain uniform dispersion.

**Table 1:** Materials and their respective quantities

Material	Percent (v/v or w/v)	Amount (ml or g)
CS	70% v/v	80 ml
β-TCP	21% w/v	15.8 g
nHA	6% w/v	7.2 g
Postbiotic	3% v/v	2.4 ml

After all materials were combined, the mixture underwent 1.5 h of sonication to remove any remaining air bubbles. For the porous foam form, the mixture was poured into cylindrical plastic molds, frozen at − 4 °C for 24 h, and then subjected to freeze-drying (lyophilization) using a laboratory freeze-dryer at the same temperature. Finally, the composite was sterilized with ethylene oxide. To obtain a paste formulation, the freezing and lyophilization steps were omitted.

### Characterization of the composite

Functional groups of the adhesive composite were identified using Fourier transform infrared spectroscopy (FTIR) with a Tensor II spectrometer (Bruker, Germany) in the range of 400–4000 cm⁻¹. The crystalline structure and phase composition of the adhesive composite were analyzed using X-ray diffraction (XRD) with an ASENWARE AW-XDM300 diffractometer (ASENWA, China). Measurements were performed with Cu Kα radiation (λ = 1.54184 Å) at 40 kV and 30 mA, scanning over a 2θ range from 0° to 60° with a step size of 0.05° and a step time of 1 s. Surface area, pore volume, and pore size distribution of the freeze-dried composite were evaluated using Brunauer–Emmett–Teller (BET) analysis with a NOVA automated gas sorption system (Quantachrome Instruments, USA) at 77.35 K. Prior to measurement, approximately 0.1 g of the sample was degassed under vacuum following standard protocols. Nitrogen gas (N₂) with a molecular cross-sectional area of 16.2 Å² was used as the adsorbate. Field emission scanning electron microscopy (FESEM) coupled with energy dispersive X-ray spectroscopy (EDS) (Sigma 300-HV, Zeiss, Germany) was used to examine the surface morphology and elemental composition of the adhesive composite. The compression mechanical properties of the adhesive foam were evaluated using a universal testing machine (Zwick/Roell, Germany) equipped with a 250 N load cell. Cylindrical specimens (6.5 mm in diameter × 12 mm in height) were compressed at a crosshead speed of 0.5 mm/min at room temperature (23 ± 2 °C) until 50% strain. Load–displacement data were recorded, and the ultimate compressive strength (UCS) was calculated as the maximum stress prior to failure. The compressive modulus (Young’s modulus) was approximated from the initial linear region of the load–displacement curve, taking into account the specimen geometry. Measurements were performed on three composite foam specimens.

### Animal model and experimental design

This in vivo study was conducted on 28 male Sprague–Dawley rats (12 weeks old; 220 ± 20 g) [24], randomly assigned to four experimental groups. The study protocol was approved by the Institutional Animal Ethics Committee (Approval Code: IR.SUMS.AEC.1403.031) and complied with international guidelines for the care and use of laboratory animals.

**Fig. 1:**
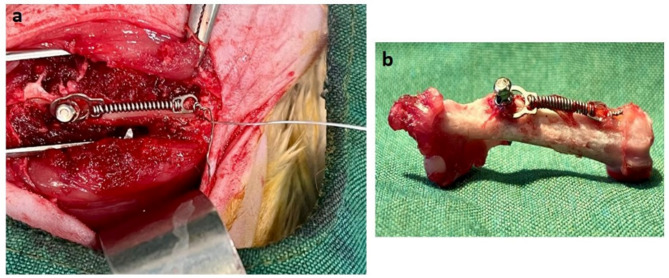
Closed coil spring ligated between the small hole and orthodontic miniscrew to apply orthodontic force. **a** During surgery. **b** After resection of the femurs

All surgical procedures were performed under general anesthesia induced by intraperitoneal injection of ketamine (60 mg/kg) and xylazine (10 mg/kg) (Alfasan, Netherlands). A titanium–vanadium alloy orthodontic miniscrew (F-type; 1.02 mm diameter, 6 mm length; MEDEFF, Iran) was inserted into the left femur of each rat under sterile conditions. A 0.6 mm hole was drilled 15 mm distal to the miniscrew site. To simulate orthodontic loading, a 100 g Ni–Ti closed coil spring (6 mm length; YaHong, China) was placed between the miniscrew and the hole. A sterile ligature wire was passed through the drilled hole and tied securely to one end of the spring, while the other end of the spring was ligated to the miniscrew head, as shown in Fig. 1. This setup was used during both primary insertion and subsequent reinsertion.

Postoperative care included intramuscular flunixin (2.5 mg/kg) for 3 days and enrofloxacin (5 mg/kg) for 7 days to manage pain and prevent infection, respectively.

After 4 weeks, six randomly selected rats (Group I) were euthanized via carbon dioxide inhalation to assess the stability of primary insertion of the miniscrews. The remaining 22 rats underwent a second surgical procedure, during which the initial miniscrews were carefully retrieved by a reverse turn of the screwdriver. These rats were then randomly allocated into three reinsertion groups as follows:

- Group II (*n* = 6): Miniscrews were reinserted into the original sites without any adhesive.
- Group III (*n* = 8): Defect sites were filled with a paste-form adhesive before miniscrew reinsertion.
- Group IV (*n* = 8): Defect sites were filled with a foam-form adhesive before miniscrew reinsertion.

The slightly larger sample sizes in Groups III and IV were intended to account for the potential risk of animal loss due to additional interventions involving adhesive materials, thereby ensuring adequate statistical power. No animal losses occurred during the study.

In each case, a new miniscrew of the same dimensions was reinserted into the original site. The orthodontic loading procedure and postoperative regimen were identical to those used during primary insertion.

Three animals from each group were randomly selected for histomorphometric analysis, and the remainder were subjected to biomechanical pull-out testing. All rats were housed individually under controlled environmental conditions (22 ± 2 °C, 55 ± 5% humidity, 12 h light/dark cycle, 10–15 air changes/hour) with ad libitum access to food and water.

Following another 4-week period, the remaining animals were euthanized to evaluate the stability of the reinserted miniscrews.

### Histomorphometric analysis

After euthanasia, the femurs were harvested and the miniscrews were carefully removed. The specimens were fixed in 10% neutral-buffered formalin and decalcified in 10% ethylenediaminetetraacetic acid (EDTA) with continuous agitation for 8 weeks. Following decalcification, the samples were embedded in paraffin, sectioned at 5 μm, and stained with hematoxylin and eosin (H&E). Histological evaluation was performed using an Olympus light microscope equipped with a 1/2 photo adaptor. Each sample was independently analyzed twice by a single blinded examiner, and the average of the two measurements was used for analysis. Two histomorphometric parameters were assessed for each sample. The bone-to-implant contact (BIC, %) was calculated as the percentage of the screw surface directly in contact with bone tissue, measured from the outer cortical plate to the third uppermost thread embedded within the bone. The bone volume fraction (BV, %) was defined as the percentage of bone area within the region bounded by the screw surface and an imaginary line connecting the tops of the threads in the same anatomical area [25].

### Pull out test

To evaluate the mechanical stability of the miniscrews, a pull-out test was performed using a universal testing machine (Zwick/Roell, Germany). The test was conducted at room temperature (23 ± 2 °C) and executed by a single trained operator to minimize variability. After the designated periods, the femur samples containing miniscrews were placed between the clamped jaws of the machine and a tensile load was applied along the longitudinal axis of the screw at a constant crosshead speed of 0.5 mm/min until complete dislodgement from the bone. The applied force was continuously recorded throughout the procedure, and the peak force (in Newtons) required for complete screw extraction was defined as the maximum pull-out strength. This protocol was consistently applied to all experimental groups to enable reliable intergroup comparisons of biomechanical stability.

### Statistical analysis

Statistical analyses were performed using SPSS software (version 18.0; SPSS Inc., Chicago, IL, USA). The normality of the data distribution for each variable was assessed using the Shapiro–Wilk test. As the data followed a normal distribution, parametric tests were employed for further analysis. Intergroup comparisons were conducted using one-way analysis of variance (ANOVA), with the significance threshold set at *p* < 0.05. Where significant differences were detected, Duncan’s post hoc multiple range test was employed to identify specific pairwise group differences. In addition, effect sizes were calculated using Eta-squared (η²) to quantify the magnitude of differences between groups, which is particularly relevant considering the relatively small sample size in each group.

## Results

### Characterization of the composite

#### FTIR analysis

**Fig. 2:**
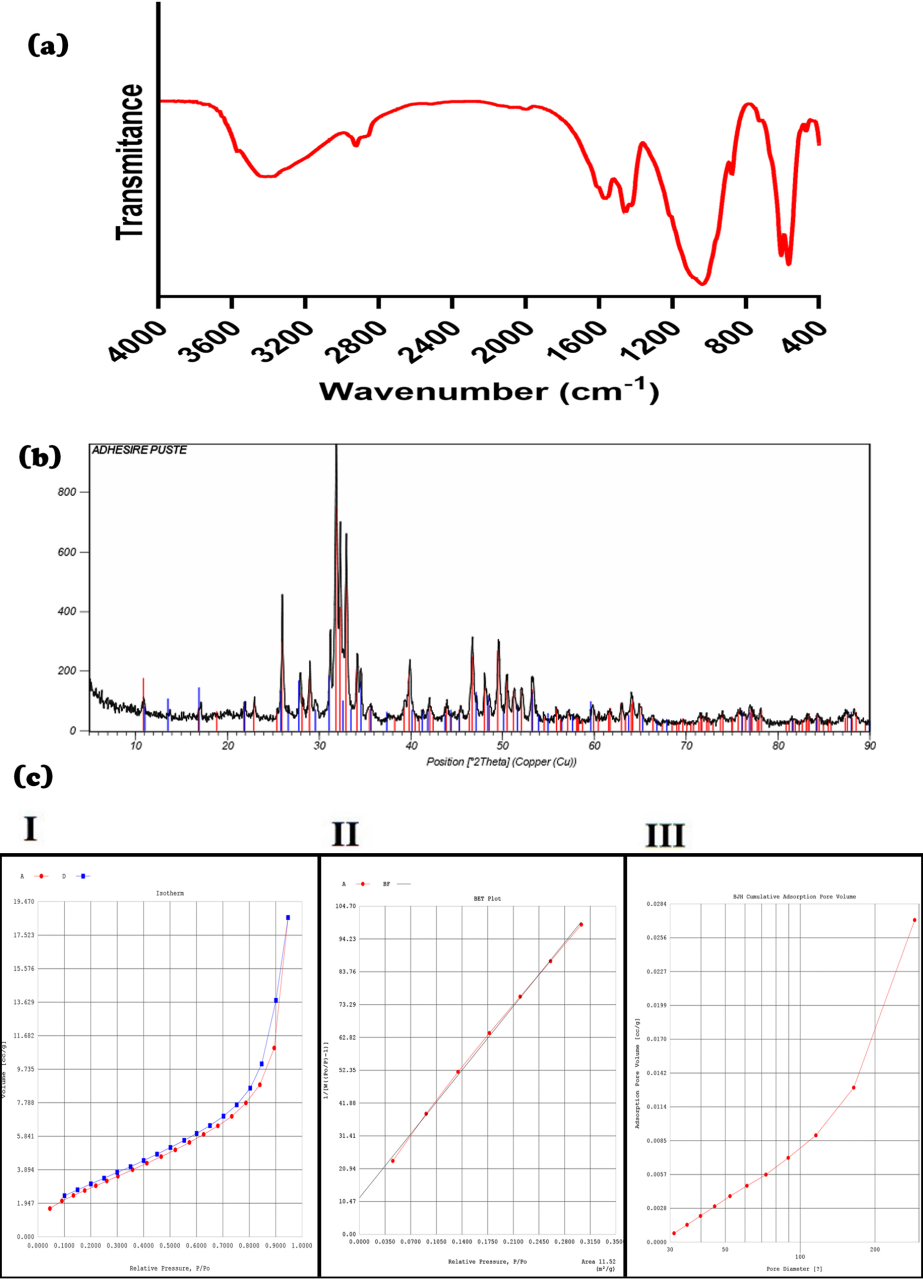
**a** FTIR spectra of the composite. **b** XRD pattern of the composite. **c** Plots. (I) Nitrogen adsorption‒desorption isotherms (linear scale). (II) BET plot for surface area determination. (III) BJH cumulative adsorption pore volume distribution

The FTIR spectrum of the composite adhesive (Fig. 2a) demonstrated characteristic peaks corresponding to its major constituents. The broad band around 3400 cm⁻¹ indicated O–H and N–H stretching from chitosan and hydroxyapatite. Peaks between 900 and 1200 cm⁻¹ were attributed to P–O stretching, confirming the presence of phosphate groups from HA and β-TCP. Amide I (1600–1660 cm⁻¹) and amide II (1550–1560 cm⁻¹) bands supported the inclusion of chitosan. C–H stretching vibrations (2870–3000 cm⁻¹) suggested the presence of postbiotic organic components. The region near 560–600 cm⁻¹ corresponded to O–P–O bending in the ceramic phases [20, 26, 27]. These results indicate the presence and interaction of the intended components within the composite.

#### XRD analysis

The XRD pattern of the composite (Fig. 2b) confirmed the presence of two crystalline phases: hydroxyapatite (HA; Ca₅ (PO₄)₃(OH), PDF 01-073-0294) and β-tricalcium phosphate (β-TCP; Ca₃ (PO₄)₂, PDF 00-006-0426). HA appeared as the dominant phase (score: 70; scale factor: 0.711), while β-TCP was present as a secondary phase (score: 33; scale factor: 0.127). Sharp, intense peaks of HA indicates high crystallinity, likely due to its nanostructure and small crystallite size. Broader and less intense peaks of β-TCP may suggest partial amorphous content. Chitosan was not detected, consistent with its known amorphous nature [28–30]. The incorporation of ceramic phases within the biopolymeric matrix was confirmed, demonstrating the formation of a composite structure.

#### BET analysis

BET surface area analysis demonstrated that the foam had a specific surface area of 11.52 m²/g and a total pore volume of 0.0285 cm³/g. The nitrogen adsorption–desorption isotherm (Fig. 2c-I) exhibited a Type IV curve with minimal hysteresis, indicative of a mesoporous structure. The BET plot was linear in the relative pressure range of 0.05–0.35 (Fig. 2c-II), confirming model suitability. Barrett‒Joyner‒Halenda (BJH) pore size distribution analysis (Fig. 2c-III) revealed a dominant pore diameter of ~ 3 nm.

##### FESEM and EDS

**Fig. 3:**
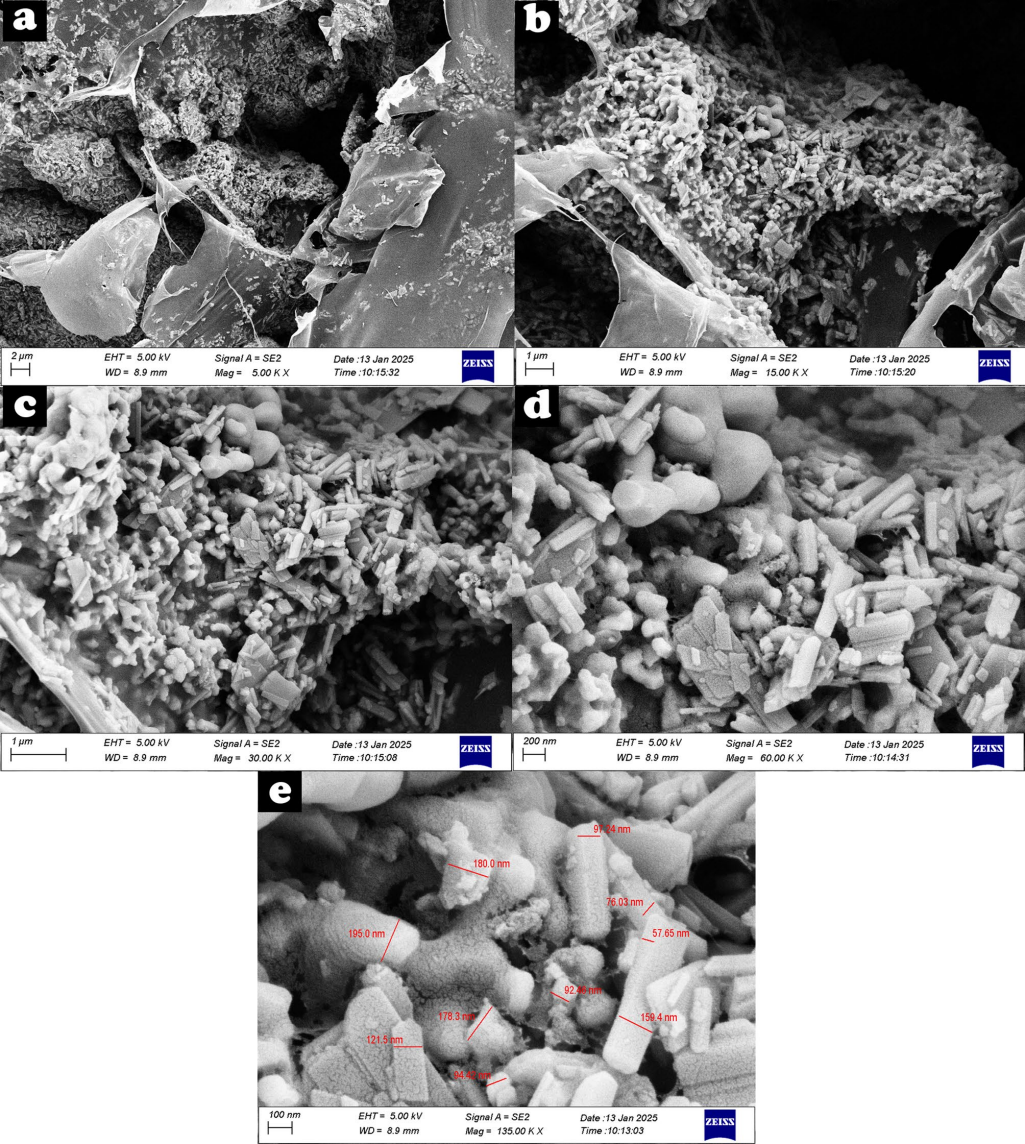
FESEM images of the foam adhesive. **a** and **b** High-density particles in the matrix. **c** Uniform distribution of n.HA and β-TCP within the polymer matrix. **d** Porous architecture. **e** Particle size distribution

FESEM imaging (Fig. 3a–d) revealed a porous microstructure with uniformly distributed ceramic particles (nHA and β-TCP) within the chitosan matrix. The porosity resulted from solvent sublimation during freeze-drying, creating an interconnected structure suitable for fluid infiltration and cell migration. Minor particle agglomerations were observed, likely due to incomplete sonication, suggesting a need for optimization. Particle size ranged from 57.6 to 195.0 nm, with an average of 125.2 nm, consistent with bioactive nanomaterials. (Fig. 3e).

**Fig. 4:**
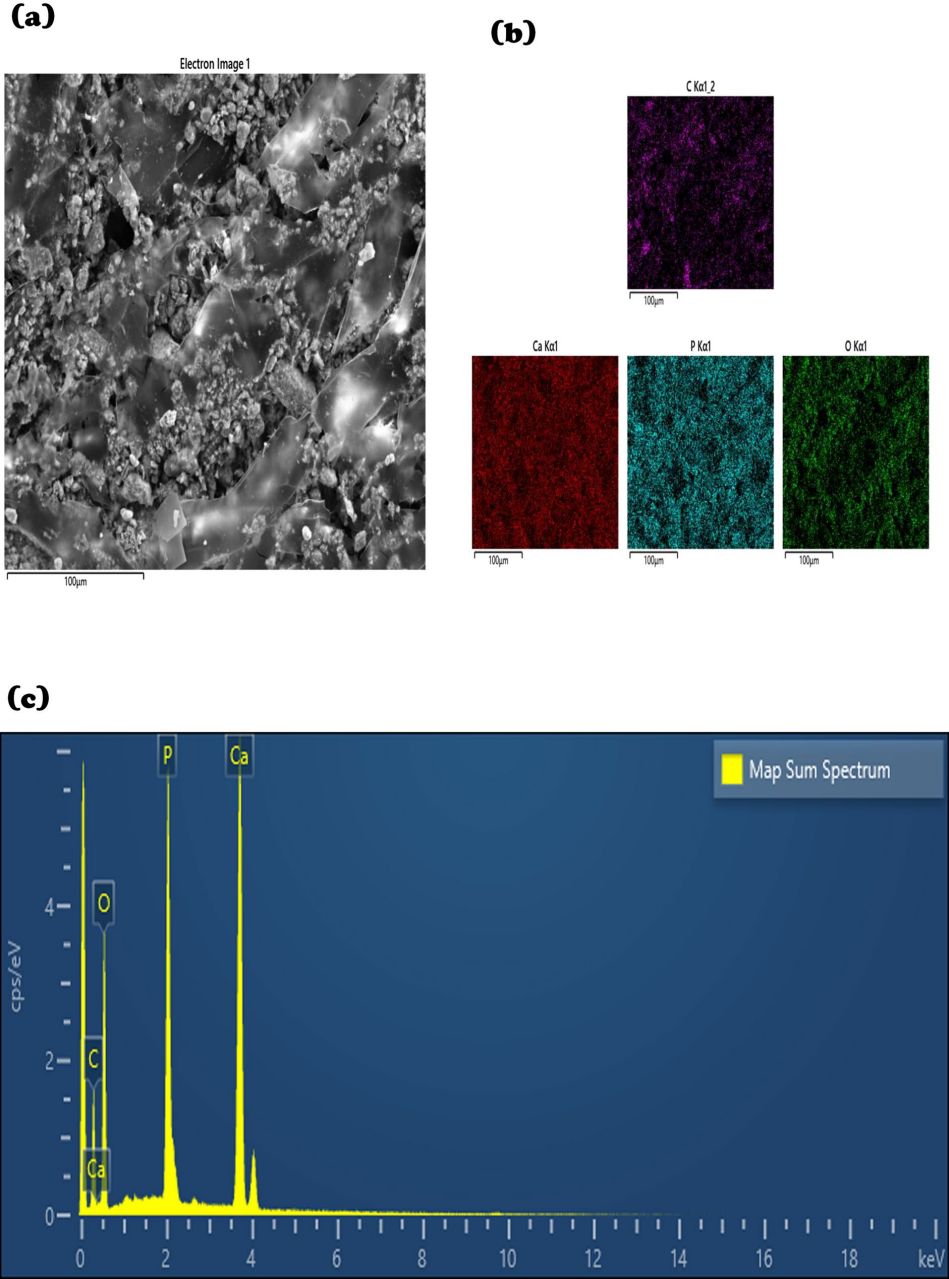
**a** Electron image and EDS image of the elemental distribution in the adhesive foam. **b** Uniform distribution of elements in the adhesive foam. **c** EDS spectrum of the synthesized foam. The spectrum displays the characteristic peaks of carbon (C), oxygen (O), phosphorus (P), and calcium (Ca), confirming the incorporation of n.HA *and β-TCP*

EDS mapping (Fig. 4a–c) confirmed the presence of carbon, oxygen, calcium, and phosphorus, with uniform elemental distribution. The carbon signal reflected the chitosan matrix and postbiotic content, while Ca and P confirmed the ceramic phases. The presence of uniformly distributed organic and inorganic elements indicates their effective incorporation within the composite structure.

#### Compression test

The foam demonstrated moderate compressive strength and substantial deformation at maximum load. The adhesive foam exhibited a mean ultimate compressive strength (UCS) of 0.387 ± 0.038 MPa, with a corresponding strain of 0.406 ± 0.088. The average Young’s modulus was 0.972 ± 0.147 MPa. Variations among specimens were minimal.

#### Histological and histomorphometric analysis

##### BIC and BV

The minimum, maximum, and mean values of BIC and BV for each experimental group are presented in Table 2. Although the adhesive-treated groups (paste and foam) showed higher mean BIC and BV values compared to control groups, these differences did not reach statistical significance (*p* > 0.05).

**Table 2:** BIC and BV

Group	BIC			BV		
	Minimum (%)	Maximum (%)	Mean ± SD (%)	Minimum (%)	Maximum (%)	Mean ± SD (%)
Control (4 weeks)	66.28	72.16	69.52 ± 2.47 ^A^	55.42	69.42	63.36 ± 5.86 ^A^
Control (8 weeks)	67.15	75.58	71.73 ± 3.60 ^A^	59.45	68.41	62.63 ± 4.05 ^A^
Paste	71.29	78.54	74.97 ± 3.12 ^A^	66.42	72.49	69.44 ± 2.61 ^A^
Foam	66.25	78.49	73.68 ± 5.36 ^A^	64.85	72.49	68.73 ± 3.41 ^A^
P value	0.247			0.079		

BIC indicates bone‒implant contact, and BV indicates bone volume. The values are presented as the means ± standard deviations, minimums, and maximums. In the mean values column, mean values with different superscript letters are statistically significant (Duncan’s post hoc test)

Effect size analysis (Table 3) provided additional insight into the clinical relevance of the observed trends. A large effect size was observed for BIC when comparing the 4-week control group to the paste group, suggesting a potential trend toward increased early bone contact, although it was not statistically significan. The smallest effect sizes for BIC were found between the paste and foam groups, suggesting minimal difference between these two adhesive treatments. For BV, large effect sizes were identified in pairwise comparisons between adhesive-treated and control groups, indicating a meaningful increase in peri-implant bone volume associated with the adhesives. Notably, the 4-week versus 8-week control comparison and the paste versus foam comparison yielded small effect sizes. This suggests minimal differences in bone volume between these specific group pairs.

**Table 3:** Effect sizes for the BIC, BV and PS

	BIC (f)	BV (f)	PS (f)
Control (4 weeks) vs. Control (8 weeks)	0.2053	0.0576	0.6313**
Control (4 weeks) vs. Paste	0.5085**	0.4862**	0.8293**
Control (4 weeks) vs. Foam	0.3881*	0.4294**	2.0208**
Control (8 weeks) vs. Paste	0.3032*	0.5438**	1.5350**
Control (8 weeks) vs. Foam	0.1829	0.4870**	2.7265**
Paste vs. Foam	0.1204	0.0568	1.3758**

BIC, bone‒implant contact; BV, bone volume; PS, pullout strength. Effect sizes were calculated using Eta-squared (η²) and converted to Cohen’s f to quantify the magnitude of differences between groups. Values of less than 0.25 indicate small effects, 0.25–0.4 (*) indicate medium effects, and values greater than 0.4 (**) indicate large effects

#### Histological analysis

**Fig. 5:**
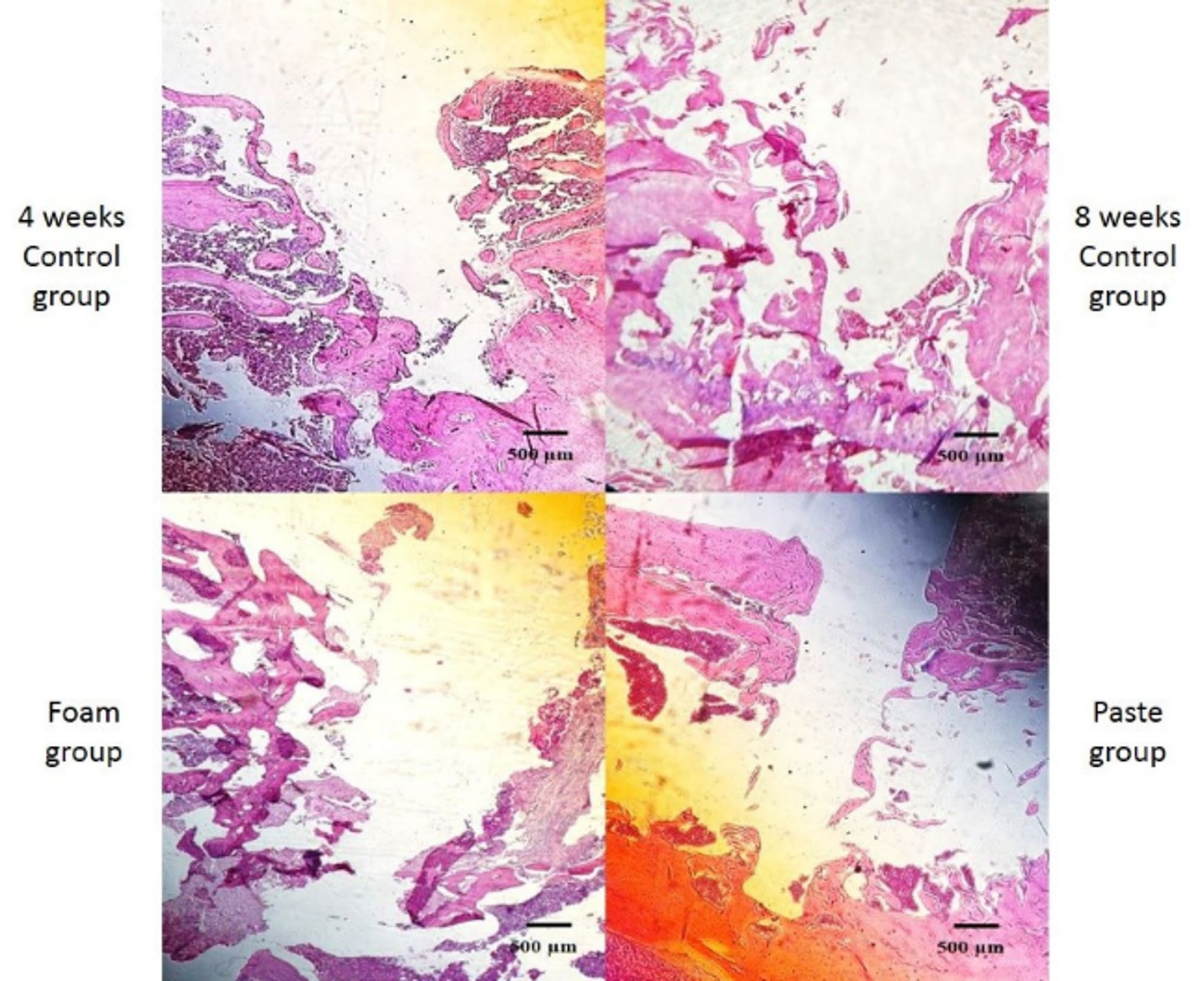
Histological micrographs of hematoxylin and eosin staining around the inserted orthodontic miniscrew (original magnification ×4)

**Fig. 6:**
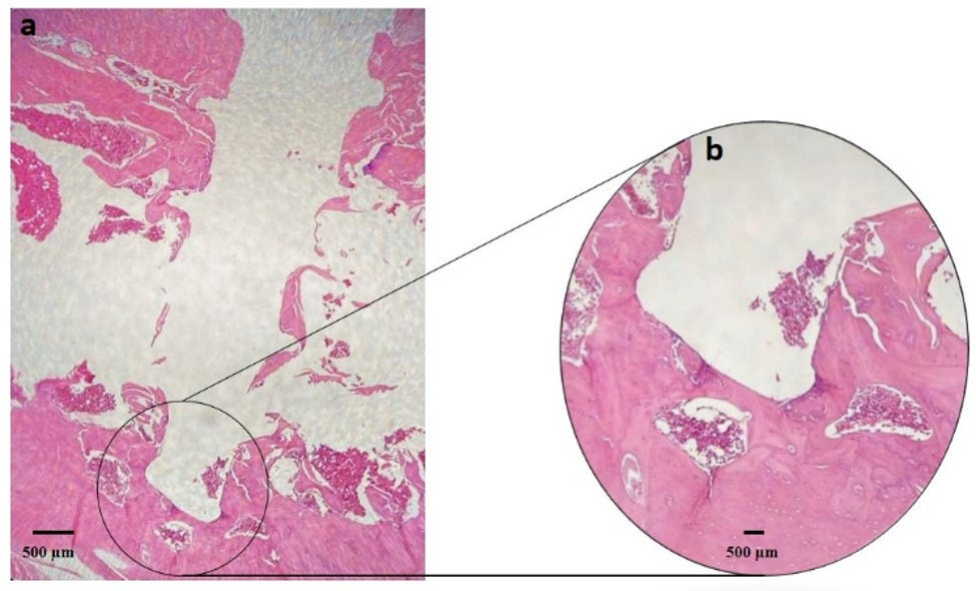
Histological micrographs of hematoxylin and eosin staining around the inserted orthodontic miniscrew in the paste group. **a** Original magnification ×4, **b** The tip of inserted miniscrew (original magnification ×10) demonstrate a greater density of osteocytes adjacent to the implant

Histological examination revealed no signs of infection or necrosis in any of the groups. The 8-week control group exhibited evident tissue and bone degradation surrounding the miniscrew site, indicating impaired healing. In contrast, the 4-week control group presented more organized tissue architecture with less evidence of degradation. The foam-treated group showed granulation tissue with early cartilage islands, consistent with an active reparative phase. The paste-treated group exhibited bone-like tissue formation with early trabecular structures, suggesting more progressed osteogenesis and closer interaction with the miniscrew surface (Fig. 5). While the foam group showed features consistent with an early reparative phase, the paste group demonstrated signs of more advanced bone-like tissue organization (Fig. 6).

### Pull-out strength

The pull-out strength results are summarized in Table 4. One-way ANOVA revealed significant differences among the groups (*p* < 0.001). Post hoc analysis using Duncan’s multiple range test demonstrated that both adhesive-treated groups (foam and paste) exhibited significantly higher pull-out strength compared to the control groups at 4 and 8 weeks. Notably, the foam group achieved the highest mean pull-out strength, whereas the 8-week control group showed the lowest. Effect size calculations (Table 3) supported these findings, showing large effects for most pairwise comparisons between adhesive-treated and control groups, especially for the foam group, suggesting that the adhesive composites may enhance miniscrew retention.

**Table 4:** PS

Group	Minimum (*N*)	Maximum (*N*)	Mean ± SD (*N*)
Control (4 weeks)	45.0	56.0	50.43 ± 5.50 ^A^
Control (8 weeks)	33.7	41.4	38.37 ± 4.10 ^B^
Paste	58.4	69.1	64.60 ± 4.50 ^C^
Foam	77.3	97.7	84.96 ± 8.34 ^D^
P value	< 0.001		

PS indicates the pull-out strength (N, Newtons). The values are presented as the means ± standard deviations, minimums, and maximums. One-way ANOVA revealed a statistically significant difference in pull-out strength among the groups (p value < 0.001). In the mean values column, mean values with different superscript letters are statistically significant (Duncan’s post hoc test)

## Discussion

This animal study aimed to investigate a novel, biocompatible composite material in the context of immediate same-site reinsertion of a new miniscrew after failure—targeting situations with a higher risk of early loosening rather than routine primary placement or long-term retention. The rationale for immediate reinsertion and reloading in this study was to simulate a clinically relevant situation in which treatment delays are undesirable. Post-failure reinsertion of mini-screws faces several challenges, including the required healing period for reinsertion at the same site, anatomical limitations that may prevent reinsertion at alternative locations, and comparable failure rates between reinsertion at original and different sites, which may suggest that failure is not purely site dependent and could be influenced by host-related factors such as age, sex, and oral hygiene [6, 9, 10]. These challenges restrict immediate successful reinsertion, potentially compromising treatment efficiency and patient compliance. Therefore, the development of an adhesive material that enhances primary stability and enables immediate reinsertion without a healing interval could provide a significant clinical advantage.

Align with previous clinical reports indicating lower success rates for reinserted orthodontic miniscrews [31, 32]; in our model, secondary insertions without adhesive exhibited significantly reduced stability relative to primary insertions. This trend parallels the results of Uesugi et al. [6], who observed a success rate of only 44.2% for secondary insertions versus 80.4% for primary placements, reinforcing the clinical challenges of managing failed miniscrews and the potential benefit of adjunctive materials.

Although biological (secondary) stability is achieved in successful miniscrews through bone remodeling, true osseointegration is generally not required due to the temporary nature of miniscrew application. As such, their stability primarily depends on the mechanical interlocking of the screw threads within the surrounding cortical bone [31, 33], typically assessed through histomorphometric parameters such as bone-to-implant contact and bone volume [34, 35]. Although differences in BIC and BV between the study groups did not reach statistical significance (*p* >0.05), effect size analysis indicated a large difference in BV between the control and adhesive-treated groups. This finding suggests a potential enhancement in local bone regeneration associated with the bioactive composite. These clinically relevant results, possibly masked by the limited sample size, are in line with the results of Nishioka-Sakamoto et al., who reported increased BV and bone mineral density (BMD) at β-TCP-treated reinsertion sites in a rat model [8]. Moreover, in both studies, histological evaluation revealed a greater presence of new bone-like tissue in the biomaterial groups, supporting the regenerative potential of such bioactive formulations. Interestingly, contrasting outcomes were reported in a study using OsteoCrete during initial miniscrew placement in the jawbone of beagle dogs, where no significant improvement in bone volume or stability was observed compared to controls [36]. This discrepancy may arise from differences in material composition and timing of application. OsteoCrete, primarily magnesium phosphate-based with limited osteogenic potential, was applied during the initial insertion phase, when bone remodeling is still ongoing, potentially restricting the regenerative effects of biomaterials. In contrast, during reinsertion, as investigated in the present study, the bone is at a more advanced healing stage, which may enhance its responsiveness to bioactive materials and improve their effects on both stability and bone regeneration.

Notably, in contrast to BIC and BV, the pull-out strength significantly improved in the adhesive-treated groups. This finding suggests that mechanical interlocking, rather than histological bone formation alone, plays a critical role in the early stabilization of reinserted miniscrews. Previous studies have shown that even limited bone contact (as low as 5%) can yield sufficient anchorage strength (200–300 g), highlighting the functional importance of mechanical integration, particularly during early healing stages when osseous remodeling remains incomplete [37]. Accordingly, pull-out testing, which directly measures the maximum force required to dislodge the screw, may offer a more sensitive and clinically predictive assessment of early miniscrew stability, especially in interventions involving immediate reinsertion where full osseointegration has not yet occurred [38, 39]. Prior studies, including those by Salmoria et al., have shown that pull-out testing is more effective than insertion torque in detecting differences in screw retention [40]. However, the pull-out test is inherently destructive, limiting its application to endpoint evaluations in laboratory settings [41]. Despite this limitation, it remains the most direct and functionally meaningful method for assessing primary stability.

To assess how material morphology influences the stabilization of reinserted miniscrews, this study uniquely compared two physical forms of the bioactive composite, paste and foam, while prior research with calcium phosphate-based adhesives has focused primarily on paste formulations [15, 36]. A comparative evaluation of the paste and foam formulations revealed distinct but complementary strengths. The paste demonstrated higher BIC and BV values, likely due to its cohesive consistency and close adaptation to the bone hole, which help retain bioactive agents and support improved osteogenesis. These features may make the paste particularly advantageous in procedures where osseointegration is a long-term goal, such as dental implant placement. In contrast, the foam exhibited significantly greater pull-out strength, attributable to its elastic, porous architecture, which promotes stress absorption, tissue infiltration, and early neovascularization. Compression test further confirmed that the foam is able to deform and recover under load, supporting its function as a mechanical buffer at the bone–implant interface, given that its Young’s modulus is significantly lower than that of human trabecular bone (12.1–22.2 GPa) [42]. These findings emphasize the form-dependent behavior of the composite. While the paste may promote more pronounced early histological integration, this should be interpreted in the context of the temporary nature of miniscrews rather than as permanent osseointegration; meanwhile, the foam provides superior mechanical retention under immediate loading.

The mechanical and biological performance of the bioactive adhesive composite developed in this study was directly influenced by its material composition. A 70% chitosan-based matrix was selected to achieve an optimal balance between scaffold porosity and mechanical strength. As confirmed by FESEM and BET analysis, this ratio produced a porous microstructure conducive to cell infiltration and bone ingrowth while avoiding excessive brittleness. Although increasing the HA/β-TCP content can improve compressive strength, our formulation intentionally limited the ceramic phase to preserve the elasticity and tensile integrity of the chitosan matrix. Additionally, the composite uniquely integrates postbiotics into the chitosan-ceramic scaffold to enhance its biological function and potentially improve its biocompatibility. Unlike probiotics, postbiotics consist of bioactive bacterial metabolites, such as short-chain fatty acids (SCFAs), that exert regenerative, antioxidant, and anti-inflammatory effects without the limitations of live organisms. These properties are particularly advantageous in bone healing contexts, as SCFAs like butyrate have been shown to stimulate osteogenesis and reduce bone loss in inflammatory or postmenopausal conditions [43–49].

In the present study, titanium alloy miniscrews were selected, as they represent the most commonly used type in orthodontic clinical practice, particularly in the buccal interradicular region, where failure rates are relatively high. The selected dimensions (1.02 mm × 6 mm) were specifically adapted to the anatomical constraints of the rat femur, which was chosen as the implantation site due to its adequate cortical thickness, standardized surface, and structural similarity to the mandibular bone [50]. Although this screw size is smaller than typical intraoral orthodontic miniscrews, it ensured safe placement and experimental consistency in the rat model. However, the absence of masticatory forces and oral microbiota in the femur may limit the generalizability of the results to the clinical oral environment. A healing period of 4 weeks was selected based on previous studies demonstrating that this duration is sufficient for the onset of new bone formation and measurable increases in mechanical stability, including maximum torque and pull-out resistance [39, 51]. While the tested adhesive composite exhibited favorable performance with titanium miniscrews, future studies should also assess its efficacy in conjunction with other commonly used materials, such as stainless steel. Moreover, future investigations incorporating extended observation periods are warranted to comprehensively evaluate the long-term biological response and mechanical performance of the material under in vivo conditions.

Despite the methodological rationale, this study presents certain limitations that should be acknowledged. The use of a non-oral animal model may limit clinical translatability, and the relatively small sample size may have reduced the statistical power, particularly for histomorphometric comparisons. Moreover, only a single concentration of postbiotics was tested, restricting dose-response analysis. Although biocompatibility testing was not conducted directly, the individual components and the composite have been previously validated [20, 52, 53]. It should be noted that only one miniscrew type and size was tested, and all miniscrews were actively removed by the investigators for reinsertion, which may not fully replicate clinical scenarios where miniscrews loosen spontaneously. While our findings suggest improved initial retention, potential effects on removal torque or ease of screw explantation were not assessed. Future studies should address these limitations by conducting biocompatibility tests, employing larger sample sizes, evaluating multiple concentrations, incorporating jawbone models or larger animals, extending observation periods, and assessing removal torque and ease of screw explantation. The use of noninvasive imaging modalities may further facilitate longitudinal assessment. Nonetheless, within the constraints of the current model, the findings offer preliminary evidence supporting the potential of bioactive adhesives for enhancing the temporal stability of reinserted orthodontic miniscrews.

## Conclusions

This animal study demonstrates the potential efficacy of a novel bioactive adhesive composite—comprising chitosan, nano-hydroxyapatite, β-tricalcium phosphate, and postbiotics— in enhancing the primary stability of a new miniscrew immediately reinserted into the same site following failure. While screws reinserted without adhesive showed reduced stability compared to primary insertions, the application of the composite significantly improved mechanical retention, with the foam formulation exhibiting superior pull-out strength. Histological observations especially in the paste group indicated trends toward more favorable bone responses, though these were not statistically significant and should not be interpreted as implant-like osseointegration. Within the limits of this model, the findings suggest that such adhesives may serve as a temporary adjunct to preserve anchorage continuity in cases of reinsertion without disrupting the treatment timeline. Nevertheless, further in vivo studies—especially in intraoral and human clinical settings—are essential to confirm biocompatibility, long-term safety, and the potential for translation into routine orthodontic practice.

## Data Availability

No datasets were generated or analysed during the current study.

## Abbreviations

CS: Chitosan
nHA: Nanohydroxyapatite
β-TCP: β-tricalcium phosphate
PS: Pull-out strength
BIC: Bone-implant contact
BV: Bone volume
TADs: Temporary anchorage devices
HA: Hydroxyapatite
FTIR: Fourier transform infrared spectroscopy
XRD: X-ray diffraction
BET: Brunauer–Emmett–Teller
FESEM: Field emission scanning electron microscopy
EDTA: Ethylenediaminetetraacetic acid
H&E: Hematoxylin and eosin
SPSS: Statistical package for the social sciences
ANOVA: One-way analysis of variance
BJH: Barrett‒Joyner‒Halenda
EDS: Energy-dispersive X-ray spectroscopy
BMD: Bone mineral density
SCFAs: Short-chain fatty acids
